# Ultra-short laser processing of 3D bioceramic, porous scaffolds designed by freeze foaming method for orthopedic applications

**DOI:** 10.3389/fcell.2024.1447979

**Published:** 2024-08-22

**Authors:** Albena Daskalova, Matthias Ahlhelm, Liliya Angelova, Emil Filipov, Georgi Avdeev, Dragomir Tatchev, Maria-Helena Fernandes, Sanjana Vig, Ivan Buchvarov

**Affiliations:** ^1^ Institute of Electronics, Bulgarian Academy of Sciences, Sofia, Bulgaria; ^2^ Fraunhofer Institute for Ceramic Technologies and Systems IKTS, Dresden, Germany; ^3^ Institute of Physical Chemistry, Bulgarian Academy of Sciences, Sofia, Bulgaria; ^4^ Faculdade de Medicina Dentaria, Universidade do Porto, Porto, Portugal; ^5^ LAQV/REQUIMTE, University of Porto, Porto, Portugal; ^6^ Physics Department, Sofia University “St. Kliment Ohridski”, Sofia, Bulgaria

**Keywords:** ultra-short laser structuring, 3D ceramic scaffolds, freeze foaming, orthopedic applications, additive manufacturing, hierarchical porosity

## Abstract

Bone substitutes are widely employed for applications in orthopedic surgery for the replacement of injured bone. Among the diverse methods that are used to design 3D bioceramic matrices, Freeze Foaming has gained attention, since it provides the ability to tune the shape of the created structures. One of the major problems related to these constructs is the lack of porosity at the outwards sides (holder) of the scaffold, thus reducing the cellular affinity and creating a rejection of the implant. In this research, we aimed to develop a bone scaffold with enhanced surface properties and improved cellular affinity. The main aim was to alter the biocompatibility characteristics of the 3D bioceramic constructs. We have produced three-dimensional, complex-shaped hollow shell structures, manufactured by Additive Manufacturing processes and as a second step, filled with a ceramic suspension by the Freeze-Foaming process. 3D constructs from HAP-derived TCP and TCP/ZrO_2_ were synthesized by freeze-foaming method and subsequently irradiated with a fs-laser (λ = 800 nm) spanning a range of parameters for achievement of optimal surface processing conditions. The designed scaffolds demonstrated enhanced topographical properties with improved porosity examined by SEM, EDX, and 3D profilometry after laser treatment. Wettability and computer tomography (CT) evaluation was also performed. The results from X-ray diffraction (XRD) and micro-Raman analysis did not show photochemical and surface or volume defects and changes after laser processing of the ceramic samples. Preliminary results from MG-63 osteoblast-like cell tests showed good cell affinity on the processed surfaces and no cytotoxic effect on the cells.

## 1 Introduction

Surface microstructuring is a common way to alter the osseointegration properties of scaffolds. It can be used to obtain surfaces with diverse characteristics using wettability, and chemistry, and thus affect the interaction process between implant and body tissue (Subia et al., 2010; Tozzi et al., 2016). Typically, the demands for biomaterials to serve as bone substitutes are complex and are expressed mainly in reliable mechanical properties, improved cytocompatibility, great extent of reliable wear resistance, and accelerated osseointegration properties (Tozzi et al., 2016; Rustom et al., 2019). The ultimate surface architecture is a challenging task to be obtained, due to the complexity of surface characteristics and biological reaction which has to be considered to obtain desired feedback in the process of implantation. Recently it has been considered that micro-, nano-engineering of biomaterial surfaces would be a suitable approach to influence scaffold bioactivity thus affecting cellular dynamics. The design of 3D scaffolds mimics the Extra Cellular Matrix (ECM) characteristics and sustains the cell’s functionality and is being extensively examined for tissue regeneration. The optimal scaffold construction should exhibit the following properties: from one side promotes nutrition provision and from the other side excrete waste products and simultaneously enable cell attachment, growth, proliferation, and dynamics (Tozzi et al., 2016; Rustom et al., 2019; Alaribe et al., 2016; Clarke, 2008). It has been studied that scaffold composition plays a major role in the function of natural tissues, however additional examinations (Subia et al., 2010; Alaribe et al., 2016) demonstrated that there is a necessity for the improvement of cellular activity by altering the surface characteristics of the designed constructs (Clarke, 2008). Ceramic materials are favorable substances for orthopedic scaffolds since they possess increased hardness, and modulated toughness (Arbez et al., 2019). Moreover, they introduce conditions, concerning inflammation development, for a more secure domain in comparison to metal biomaterials (Wang et al., 2022). A considerable number of ceramic-based biomaterial scaffolds are used in tissue engineering with the possibility to tune the shape, porosity, and dimensions, and with an opportunity to reproduce the functionality of the natural tissue. The typical structure of bone tissue is represented by a highly porous inner and very dense, mechanically stable outer composition. To mimic the hybrid bone ECM, a state-of-the-art technique, combining commercial 3D printing, and sponge-like inner bone structure foam production, by so-called Freeze Foaming was used. Freeze foaming is a method that provides porosity in the depth of the 3D biomimetic constructs and in that way ensures the interconnected microporous structure of the bone, while the additively manufactured support structure covers the load-bearing properties, needed for proper functioning of the bone scaffold (Rustom et al., 2019; Wang et al., 2022). Moreover, the combination of mechanically stable support and porous inner structure of the 3D ceramic constructs will allow inter-protection and growth of bone cells, and as a result, faster tissue recovery. By changing the surface properties, surface roughness and porosity of the scaffold could be considerably altered, thus making conditions for diverse functionalities of the scaffold, for example, smaller pore size dimensions could lead to enhanced molecular transportation and removal of waste products from cell metabolism. In contrast, larger pore dimensions would trigger cell movement and orientation (Rustom et al., 2019; Wang et al., 2022). A suitable tool for providing tunability over the fine processing of diverse biomaterial surfaces is the application of ultra-short laser pulses. Ultra-short laser-induced hierarchical surface structuring represents an alternative to conventional chemical and physical techniques since it can greatly manipulate processing parameters and very fine control of the dimensions and distribution of the created micro/nanostructures. Ultra-short laser processing in recent years has become a supplementary method to modify hard ceramic material’s surface without initiation of severe heat-affected zones and with the ability to obtain structural morphology with a high degree of geometric freedom (Carnes and Pins, 2020). The method possesses also high applicability to alter the surface roughness without residual chemical contamination leading to a change in the wettability properties (Terakawa, 2018; Zhang et al., 2018). One of the main challenges associated with laser texturing is the ability of the method to realize complex features and optimize the processing parameters. However, it is proven that the technique is capable of considerably decreasing the heat-affected zone (HAZ) (Carnes and Pins, 2020; Lenzner et al., 2000; Phillips et al., 2015). Studies have been already realized on the laser processing of 2D alumina ceramics (Daskalova et al., 2020; Han et al., 2021; Hong et al., 2022; Roitero et al., 2017), and minor analysis has been performed to systemic research of the influence of the laser process parameters on producing microstructures with a high degree of precision on 3D complex ceramics for altering surface biomimetic properties of the support structures. The application of ultra-short laser radiation for surface modification of complex 3D constructs is a newly arising approach, and its potential application in the treatment of complex 3D ceramics has to be explored.

In this study, a foaming method and an additive manufacturing method to synthesize a 3D scaffold from HAP-derived TCP and the composition of TCP/ZrO_2_ are used. Those customizable composite scaffolds are the result of the proven attempt to manufacture complex bioceramic hybrid structures that are a combination of load-bearing support and porous cell-ingrowth-allowing interior that permits the manufacturing of bone-like mechanically stable implants that, potentially, are applicable for long-bone defects (Ahlhelm et al., 2021). To be able to reproduce bone architectures with different structural sections (e.g., *substantia spongiosa* and *substantia corticalis*), which could be used as implants in the future, two technologies were recently combined. Either an outer shell of an artificial bone (Ahlhelm et al., 2017; Ahlhelm et al., 2016) or a load-bearing support structure (Ahlhelm et al., 2021) was produced using a commercial three-dimensional (3D) additive manufacturing (AM) device, and the sponge-like inner bone structure was reproduced by a ceramic foam. For foam production, so-called Freeze Foaming was used which utilizes a freeze dryer for reducing the ambient pressure around an aqueous ceramic suspension, which in turn, causes the suspension to first foam and then freeze. Ongoing pressure reduction lets the frozen water sublimate (evaporation without becoming liquid). A subsequent heat treatment produces a solid ceramic foam. As a next step, the porous bone-like structures can be fitted to a customized, complex outer ceramic shell or inner-lying support structure and, thereby, are made mechanically more stable. This is where the AM comes into play. One of the best-known processes in AM is the conventional stereolithography (SLA) process. This process allows photopolymerizable suspensions, which are filled with ceramic particles, to be cured by a UV laser. Today, the commercially available material portfolio using lithography-based ceramic manufacturing (LCM) for high-performance components also works with β-tricalcium phosphate (β-TCP) which plays a role in this contribution. The LCM technology as a projection-based (PSL) top-down process with a light source in the blue range (452–465 nm) is representative of the so-called Ceramic Additive Manufacturing Vat Photopolymerization (CerAM VPP) process. This allows a digital micro-mirror unit, which splits a light beam into individual pixels and then projects a digital image pixel-by-pixel onto the building platform. This makes it possible to image the entire contour of the component cross-section without a mask. Thus, layer by layer, a complex 3D structure is created. In the last hybridization step, the two methods–CerAM VPP and Freeze Foaming–are combined to produce porous-dense, graded, structural hybrids by a joint sintering process. Especially by choosing the path of not only foaming within additively manufactured structures but foam them in, it is possible to provide a porous and sponge-like scaffold as the lead structure for cells to grow into, and at the same time, AM parts serve as load-bearing support structures.

We aimed to use ultra-short laser texturing to assess the surface properties of complex 3D HAP-derived TCP and TCP/ZrO_2_ constructs by inducing control over the roughness and wettability characteristics of the support structures of the designed scaffolds. In our research, state-of-the-art freeze-foamed matrices are developed with a support structure. One part of the designed samples that can be regarded as a disadvantage, in terms of providing the best biointegration, is the support structures, where the cell affinity is monitored to be strongly reduced as was shown in (Ahlhelm et al., 2021), by designing additional hierarchical structures on the surface of the holder structure, via application of fs laser-based patterning, the initial surface roughness is strongly altered, thus influencing the roughness and wettability properties of the support structures and leading to cell-friendly environment.

## 2 Material and methods

### 2.1 Freeze foaming process

As raw material for the Freeze Foaming, hydroxyapatite (HAP) (Sigma-Aldrich, now Merck KGaA, Darmstadt, Germany; BET = 70.01 m^2^/g, d_50_ = 2.64 µm) was chosen. Before suspension making, it was calcined at 900°C for 2 h to reduce the BET (now 5.9 m^2^/g). The resulting ceramic suspensions are made of water, Dolapix CE 64 (Co. Zschimmer & Schwarz Mohsdorf GmbH & Co. KG, Burgstädt, Germany) as dispersing agent, the bioceramic powder, polyvinyl alcohol (PVA) as binder component and rheological modifier Tafigel AP15 (Co. Münzing Chemie GmbH, Heilbronn, Germany) in combination with 2-Amino-2-methyl-1-propanol—AMP (Merck KGaA, 64,271 Darmstadt, Germany) for pH adjustment. The following processing route was elaborated: 49 wt% deionized water, 1.3 wt% PVA binder, hydroxyapatite, and 4.6 wt% dispersing agent referring to powder content, were mixed in a centrifugal vacuum mixer (ARV310, Thinky Corporation, Fukuoka, Japan). For dispersing the particles and reducing agglomeration, the mixture was exposed to high stirring rates (2,000 rpm, mixing time 1 min, with 3 ZrO_2_ mixing spheres of 10 mm diameter). The spheres were then separated. 1.9 wt% rheological modifier together with 1.5% wt. AMP was added. For distributing the modifier, the suspension was further mixed for 2 min at 1,500 rpm. As the last step, the suspensions were filled into specific molds (see Section 2.3) and transferred to a freeze dryer (Lyo Alpha 2-4, LSCplus, Co. Martin Christ Gefriertrocknungsanlagen GmbH, Osterode, Germany) for Freeze Foaming.

### 2.2 Ceramic Additive Manufacturing Vat Photopolymerization - CERAM VPP

**FIGURE 1:**
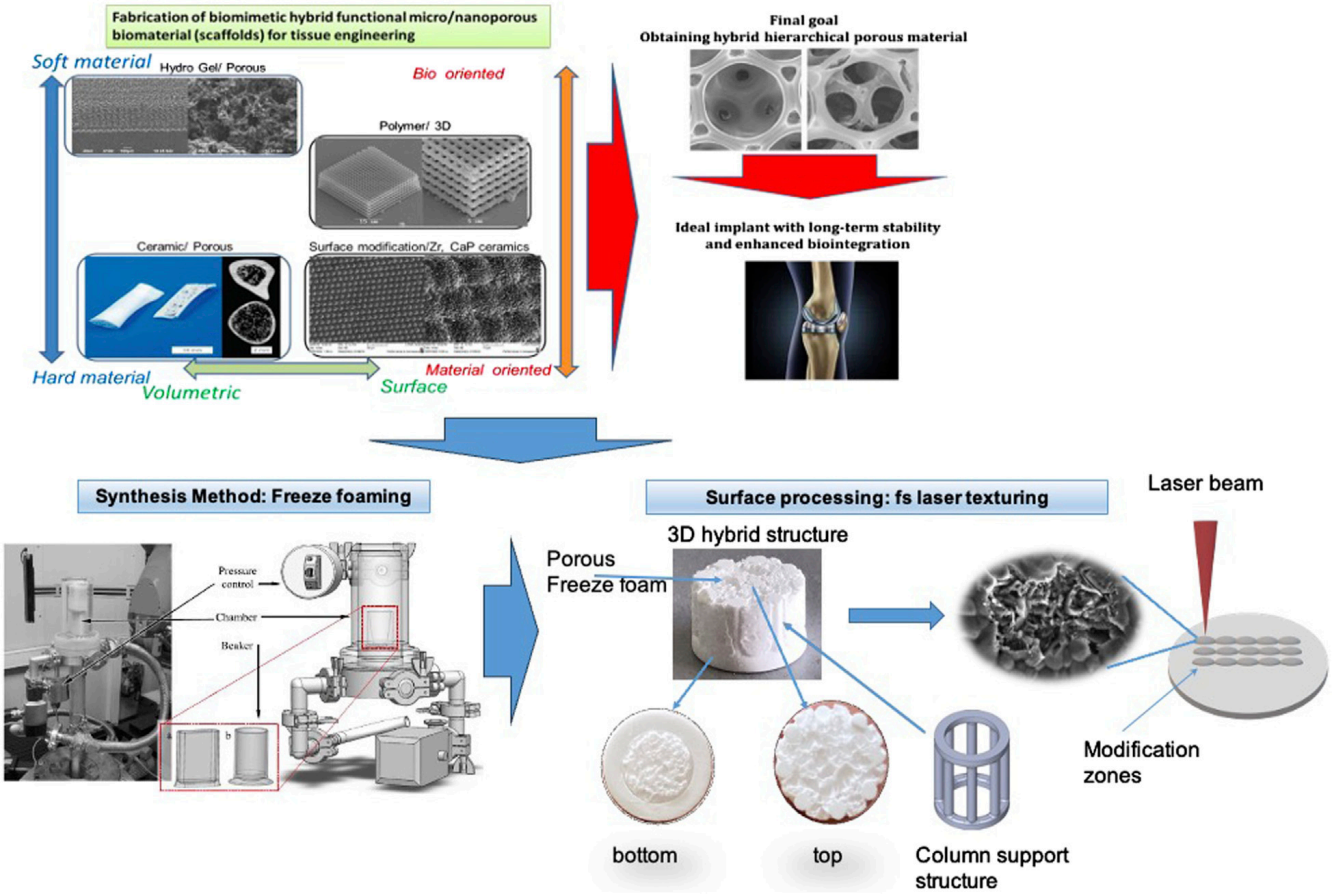
3D hybrid ceramic construct with top and bottom view and schematic representation of the laser-structuring process of the sample’s surface.

**FIGURE 2:**
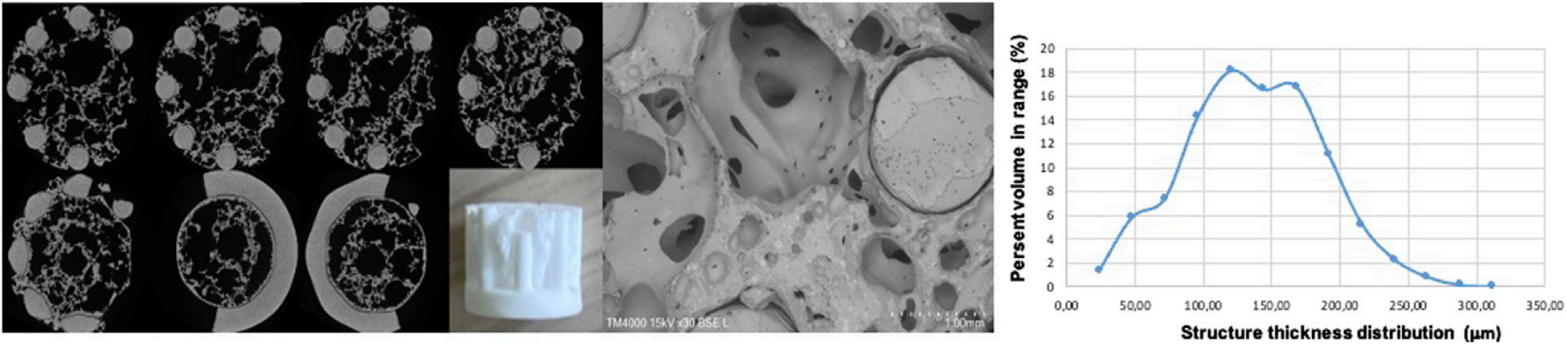
2D section images of the structure of 3D ceramic constructs, taken sequentially from top to bottom using computer tomography, SEM image of the sample and structure thickness distribution graph.

As bioceramic material for the CerAM VPP process, the same calcined hydroxyapatite was used as for the Freeze Foams. HAP powder (content: 40 vol%) was dispersed in an organic fluid (polyethylene glycol, Sigma-Aldrich, now Merck KGaA, Darmstadt, Germany) with added dispersing agent (BYK-Chemie) and various monomers that act as a binder (a mixture of acrylic resins), was well as a photoinitiator (combination of a camphor derivative and an amine). For the stepwise preparation of the suspensions (three times 5 min at 2,000 rpm) a planetary centrifugal high-speed vacuum mixer (Thinky ARV310, Thinky Corporation, Tokyo, Japan) was used. Following CerAM VPP manufacturing, the column geometry as seen in Figures 1, 2 was chosen.

### 2.3 Mold filling, hybridization and part characterization

For achieving the hybrid parts that are to be manipulated with the laser, cylindrical rubber molds were fabricated, into which the CerAM VPP parts tightly fit. In the first step, the ceramic suspension filled the molds. Then the column structure was pushed into the cavity. Subsequently, all molds were transferred to the freeze dryer (same as above) and foamed at once. A very crucial step in creating the structural hybrids is the shrinkage adjustment of the two very different structures. At the start, various suspensions were developed consisting of different amounts of water, rheological modifier, and binders. With those suspensions, Freeze Foams were manufactured that shrank between 30% and 46% (determined by thermos-dilatometry DIL 402 C/7/G Netzsch-Gerätebau, Selb, Germany). The CerAM VPP-manufactured column structures shrank around 30% altogether. It was found though that the VPP part already shrank around 5.4% at the very beginning of the heating process (the debinding), whereas the Freeze Foam part did not. As a result, the Freeze foam would have shrunk onto the VPP part, leading to a part failure. Therefore, and to compensate for the overall shrinkage, the VPP columns were pre-sintered before being used for *in situ* Freeze Foaming. This pre-sintering amounted to a shrinkage of around 5% volume. Thus, carefully adjusted for shrinkage, the hybrid parts were sintered at 1,250 K (+50 K overheating effect) for 1.5 h. Afterward, the column-including foams were dismantled and evaluated regarding porosity and microstructure. It must be noted that, after sintering, the initial hydroxyapatite was changed to β-TCP. Among many other references reporting the transition of HAP to TCP during heat treatment, similar Freeze Foams with the same initial HA powder were analyzed via XRD in a previous work (Ahlhelm et al., 2017), showing the HAP to TCP transformation. For microstructure analysis, the resulting Freeze Foams were previously characterized by SEM (Ultra 55, Co. Carl Zeiss, Oberkochen, Germany). By measuring the height and diameter of three different foam positions of manufactured Freeze Foams and deriving the average, geometrical porosities were calculated according to (P = porosity, ρth = theoretical density, ρbulk = bulk density) Equation 1:

$$P=1\;-\;\left(\frac{\rho th}{\rho bulk}\right)$$ (1)

In addition, porosity was determined via a foam structure analysis tool based on computer tomographic images of the manufactured parts. The allocation of that 3D volumetric pore morphology information (foam cell size) was managed using VGStu-dio Max v3.0 (Volume Graphics GmbH, Heidelberg, Germany). For X-ray computed tomography, a CT-Compact (Procon X-ray, max. 150 kV power) was used, the results are presented in detail elsewhere (Ahlhelm et al., 2021). In the current study, a computer tomography (using Computed X-ray microtomography Bruker SkyScan 1272, MA, United States) was performed to evaluate the 3D porous structure of the created ceramic constructs–Figure 2. The calculated sample’s porosity is 78%. The estimated average pore size is 1.1 +/− 0.8 mm. As can be seen from the 2D sections presented, an evident porous bulk is outlined in contrast to the dense and smooth structure of the sample stabilizing holders-namely these structures are the weak link in the 3D ceramic bone scaffolds, due to strongly reduced cellular attachment. Enhanced surface characteristics are achieved in the next step employing additional hierarchical structuring via ultra-short laser processing.

The construction of the created scaffolds, contain pillar-like structures, to provide mechanical stability of the whole structure. The designed 3D scaffolds possess a column-like geometry, the material used for them was TCP/ZrO_2._

### 2.4 Experimental setup for laser processing

**FIGURE 3:**
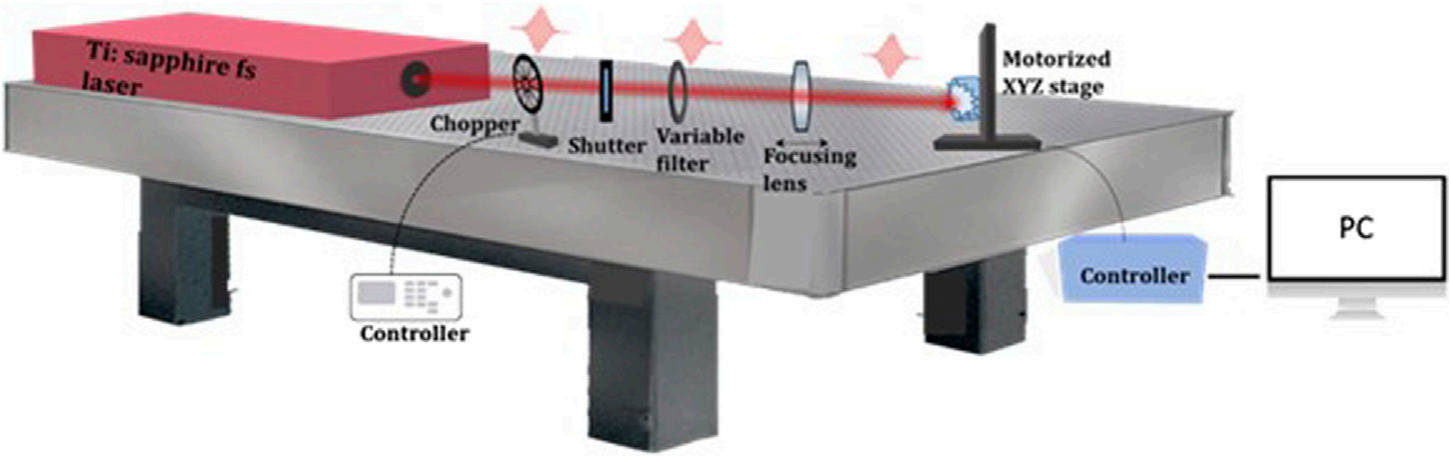
Ti: sapphire amplifier laser system Integra C experimental setup.

The schematic outline of the used laser setup is depicted in Figure 3. An ultra-short laser Ti: sapphire amplifier laser system Integra C emits highly intense pulses with a duration of (τ) = 150fs at λ = 800 nm central wavelength with a maximal pulse energy of 1 mJ. High-precision X, Y motorized translation stage allows precise sample positioning. The sample was processed in the air in line scan mode. The laser beam with a Gaussian profile was focused by a lens with a focal distance of 100 mm, which creates a focal spot with a diameter of 50 μm. The scanning speed (V) was varied between 1.7 mm/s and 32 mm/s and the fluence–in the range of F = 0.04÷0.2 J/cm^2^. A LabVIEW program enabled complete control of the scanning process and movement along the axes. After preliminary analyses, the working parameters for functionalization of scaffolds for all *in vitro* tests were chosen to be: F = 0.08 J/cm^2^ with applied number of laser pulses (N) 5. This type of modification, demonstrated most homogeneous texturing and improved porosity, which is expected to facilitate cellular adhesion.

### 2.5 Analysis of the laser-patterned 3D samples

The morphology of laser patterned samples was examined by scanning electron microscopy (SEM–Hitachi) which allows the acquisition of energy dispersive X-ray analysis - EDS (TESCAN/LYRA/XMU) to resolve the structures with high resolution and to analyze the elemental composition of the samples. SEM images at different magnifications and EDS spectra were acquired after sputtering with a nanometer layer of carbon (≈10 nm). The laser-machined 3D constructs were inspected by light microscopy and measured by confocal microscopy (Leica DCM3) using a ×50 objective lens with a stitching routine. The surface roughness profile evaluation was performed via a 3D optical profiler, Zeta-20 (Zeta Instruments, KLA, Milpitas, CA, United States), at ×20 magnification. The roughness parameters Ra and Sa were measured using 2D and 3D roughness analysis. ProfilmOnline software (https://www.profilmonline.com) was used for improved visualization of the 3D real-color reconstructions obtained. Raman analysis was acquired via a micro-Raman spectrometer (LabRAM HR Visible, Horiba, Kyoto, Japan) equipped with a microscope (BX41, Olympus, Tokyo, Japan) and a He–Ne laser working at 455 nm excitation wavelength. The Philips PW1050 X-ray diffractometer equipped with a secondary monochromator of the diffraction beam and a copper anode, having Cu Ka radiation, was used for an X-ray crystallography analysis (XRD). XRD was performed within the range of 5–70^o^ θ2 (a step size of 0.065^o^θ2 in a continuous scan mode and time of exposition-4s). The XRD spectra acquisition was performed at 40 kV and 30 mA. The phase identification was obtained via QualX2 software, through the Crystallography Open Database. Dynamic Water Contact Angle (WCA) measurements for a period of 180 s and dH_2_O at the volume of 2 μL before and after laser structuring were performed using DSA25 Drop Shape Analyzer (KRÜSS GmbH, Germany). A statistical analysis (the Mann-Whitney Test) was performed to assess whether the differences in contact angles between laser-treated and untreated surfaces were significant. Results of *p* value below 0.05 were deemed as statistically significant.

### 2.6 Effects of fs laser-treated and untreated ceramic composites on metabolic activity and morphology of MG63 cells

To assess the differential effects of laser-treated and untreated scaffolds on cells, two *in vitro* studies with osteoblast-like MG63 cells (ATCC^®^CRL-1427^™^) were carried out. One of them involved the seeding of the cells in basal media for 10 days, while for the second study, osteogenic factors were added to the basal media and the culture was continued for 7 days. The osteogenic media was chosen for cell culture in order to investigate whether the combination of surface structuring and osteogenic factors will improve osteoinduction and any potential differences in cell morphology. The basal media was composed of MEM alpha supplemented with 10% fetal bovine serum, 100 μg/mL streptomycin, 100 IU/mL penicillin, 2.5 μg/mL amphotericin B (all reagents purchased from Gibco, United States) as the cells were cultured at 37^o^C and 5% CO_2_ atmosphere. For the second experiment, the cells were conditioned with osteogenic factors for 48 h before seeding. These included 50 μg/mL ascorbic acid and 10 nM dexamethasone (Sigma-Aldrich, St. Louis, MO, United States). The seeding concentration was 2.6 × 10^5^ and upon seeding the basal culture media was supplied with 50 μg/mL ascorbic acid, 10 nM dexamethasone, and 10 mM β-glycerophosphate (Sigma-Aldrich, St. Louis, MO, United States). The seeded MG63 cells were characterized morphologically via scanning electron microscopy (SEM) as well as by the Resazurin assay for monitoring of changes in metabolism during the culture period. The morphological changes of the cells were evaluated on days 5 and 10 days of culture in basal media and on days 7 for the ones in osteogenic media. All seeded scaffolds were fixed in 1.5% glutaraldehyde solution (25% cacodylate solution used for preparation, TAAB laboratories equipment Ltd., Aldermaston, England) for 15 min and then further stored in sodium cacodylate solution. The fixed scaffolds were dehydrated by consecutive immersion in ethanol at increasing concentrations (50%, 70%, 90%, 100%). The morphological analysis was performed via scanning electron microscope (SU5000 Hitachi, High-Tech Europe) as all samples were sputter-coated with a layer of platinum (≈4 nm). All images were acquired at 15 kV. During cell culture, the metabolic activity of seeded MG63 cells was analyzed via the Resazurin assay on days 2, 5 and 9. Prior to measurements, the ceramic scaffolds with cells were relocated to a new well plate to avoid acquisition of fluorescent signal arising from cells at the bottom of the well. After that, the scaffolds were incubated with 10% Resazurin solution for 3 h (Resazurin sodium salt, Sigma-Aldrich R7017, diluted in a complete medium (alpha-MEM with 10% FBS, 100 IU/mL penicillin, 2.5 μg/mL amphotericin B, 100 μg/mL streptomycin) at 37°C. Fluorescence signal (530 nm excitation/590 nm emission) was acquired by a microplate reader (Synergy HT, Biotek, United States) using Gen5 1.09 Data Analysis Software.

## 3 Results

### 3.1 Laser ablation characteristics of 3D scaffold from HAP derived TCP and composition of TCP/ZrO_2_ ceramics composite structures

**FIGURE 4:**
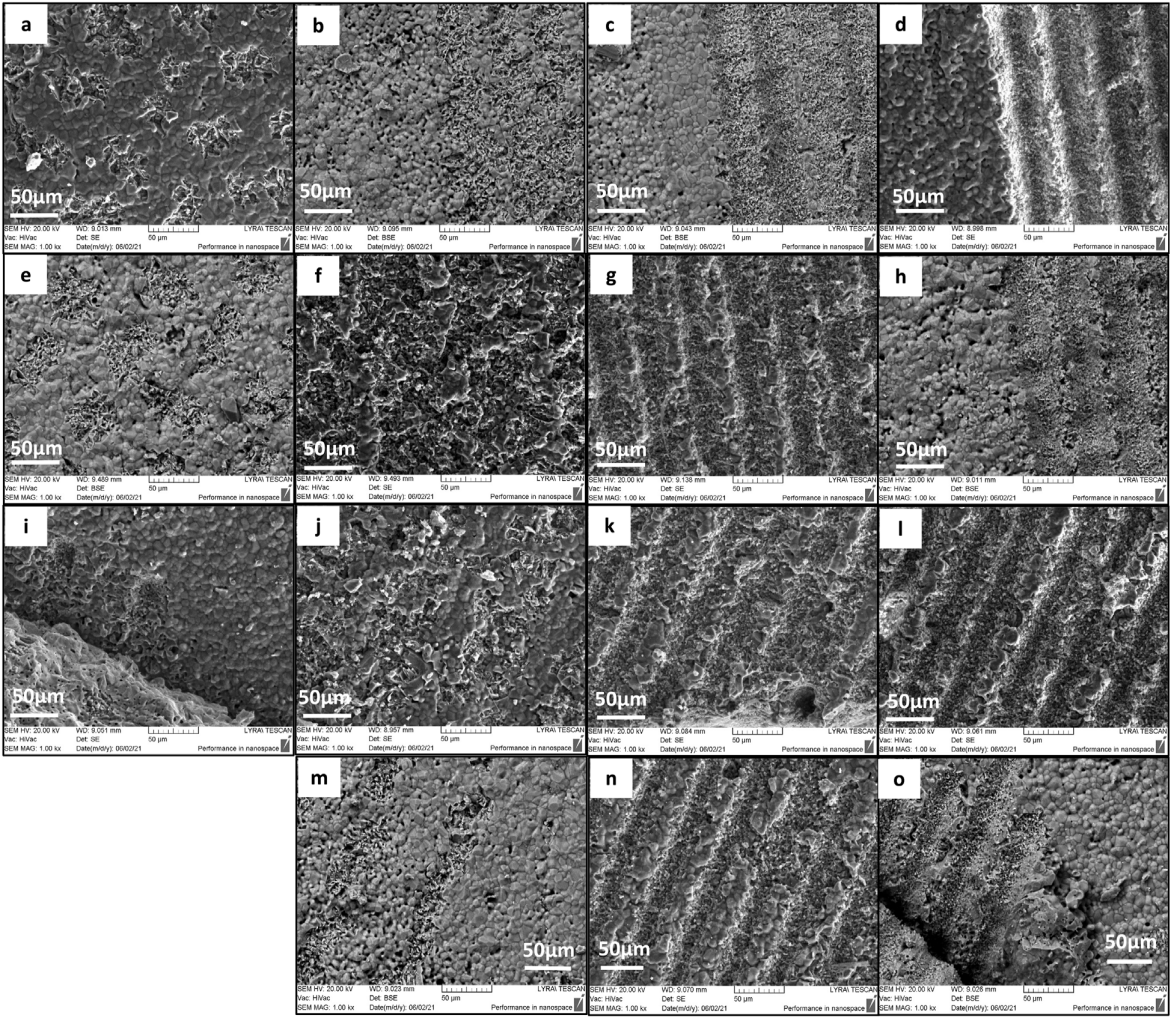
SEM images depicturing the top surface morphology of the laser processed support structures of 3D HAP derived TCP structural hybrid samples obtained under irradiation of F = 0.2 J/cm^2^; linear scan speed V = 32, 16, 3.8, 1.7 mm/s **(A–D)**; F = 0.16 J/cm^2^; linear scan speed V = 32, 16, 3.8, 1.7 mm/s **(E–H)**; F = 0.08 J/cm^2^; linear scan speed V = 32, 16, 3.8, 1.7 mm/s **(I–L)**; F = 0.04 J/cm^2^; linear scan speed V = 16, 3.8, 1.7 mm/s (**M–O)**. The pulse-to-pulse distance is optimized leading to homogeneous stripe-like formations.

In this research, novel implants with 3D design are built from additively manufactured composite ceramics - column structures foamed in with porous Freeze foam (as cell ingrowth media). To obtain microstructures on the 3D ceramic scaffolds, with a focus on the side support columns, ablation is used with exposition under a set of laser parameters. The fluence regime is tuned between 0.2 J/cm^2^ and 0.04 J/cm^2^. To alter the interaction of the laser light with matter and to obtain structuring with diverse features, several groups of surfaces were designed to study the behavior of each material concerning changes of laser fluence and scanning velocity. A qualitative SEM evaluation that unveils the top surface morphological changes of the laser-induced structures is presented in detail in Figure 4.

The laser texturing creates regularly ablated channel-like patterns at the surface of the holder. From the SEM images is possible to identify the ablation threshold (Fth) and optimal laser fluence values for processing and obtaining defined microchannels. A threshold fluence can be defined as the minimum laser fluence, which initiates material modification or ablation of the sample surface (Nathala et al., 2016). The images from SEM analysis in Figure 4 demonstrate the deviation from the initial structure morphology where structures in the shape of the microchannel are formed due to the interaction of laser light with the material, moreover, the topography constitutes from the peak-valley arrangement, which increases with laser parameter optimization. The processed surface is free from cracks, moreover, no melt flow is detected through the laser-produced traces. For the range of laser fluences (F = 0.2 J/cm^2^, 0.16 J/cm^2^, 0.08 J/cm^2^) is monitored initiation of first signs of laser-induced modification spots for scanning velocity V = 32 mm/s. The micron-sized zones are visible on the surface. At the lowest laser fluence, the detection of ablation spots was not observed. Reducing the scanning speed causes further modification of the stripes and the micro–channels became more explicit Figure 4D, I.

**FIGURE 5:**
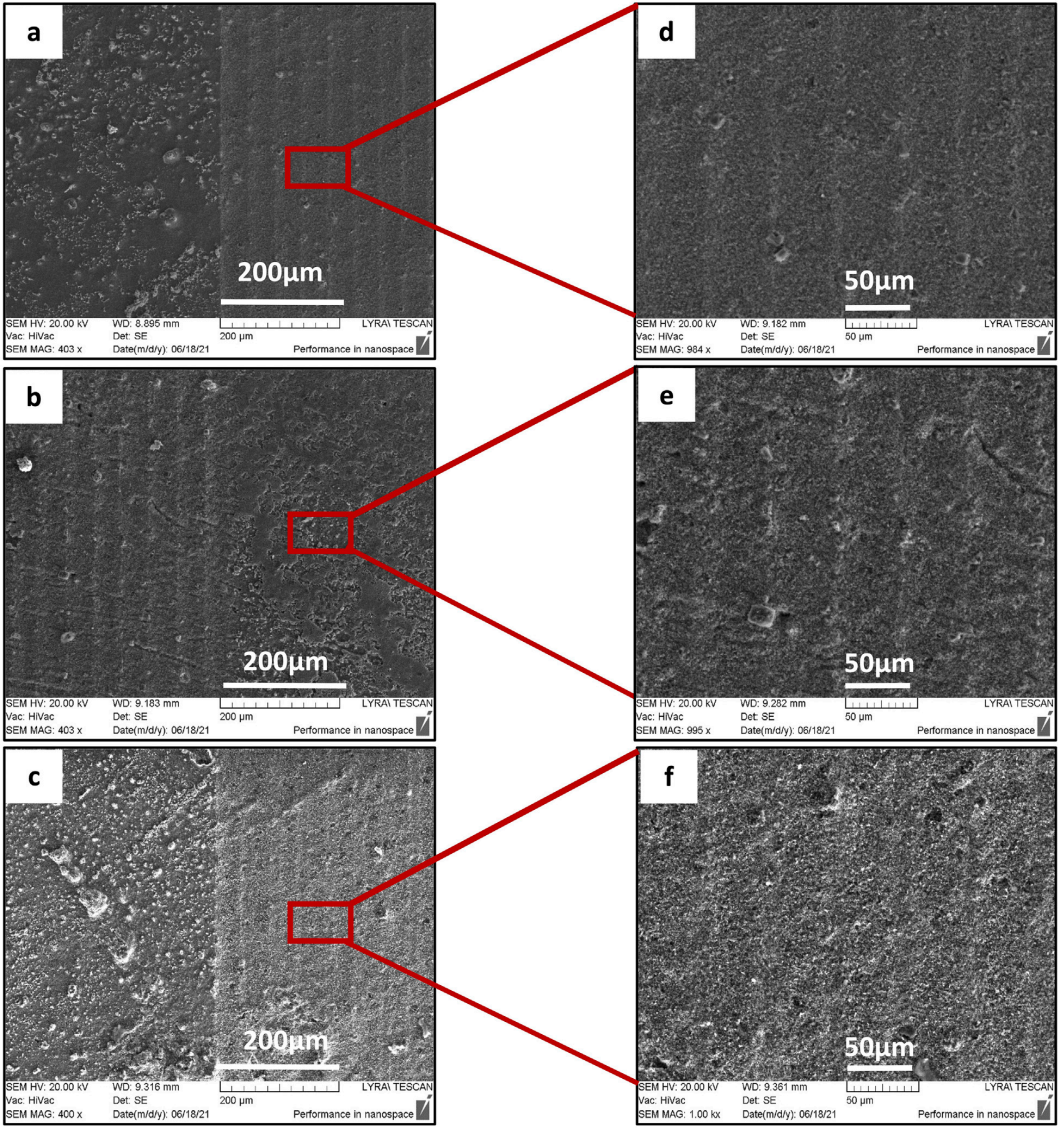
SEM images of surface morphology of 3D TCP-ZrO_2_ ceramics after laser scanning with a velocity of **(A–C)** V = 3.8 mm/s; F = 0.04 J/cm^2^, 0.16 J/cm^2^, 0.2 J/cm^2^. The right columns **(D–F)** are enlarged SEM images of the obtained patterns after laser processing.

In Figure 5, SEM images of the irradiated surface of 3D TCP-ZrO_2_ ceramic with different values of applied laser fluence at constant scanning velocity are presented.

It is observed that the increase of laser fluence at constant scanning velocity, allows the achievement of more pronounced structures, after reaching the ablation threshold. When increasing the fluence (between 0.04 J/cm^2^ and 0.2 J/cm^2^), the created lines form continuous bands without further modification of the material. For treatments with lower laser fluence, the patterned grooves start to develop. The obtained pattern profile is a result of the combination of scanning velocity and applied laser fluence. Processing the sample surface with F = 0.04 and 0.16 J/cm^2^ creates fine boundaries in contrast to morphologies obtained by increased F = 0.2 J/cm^2^. The groove-like patterns start to lose their clear limits, which could be attributed to the development of open porosities on the ceramic surface.

**FIGURE 6:**
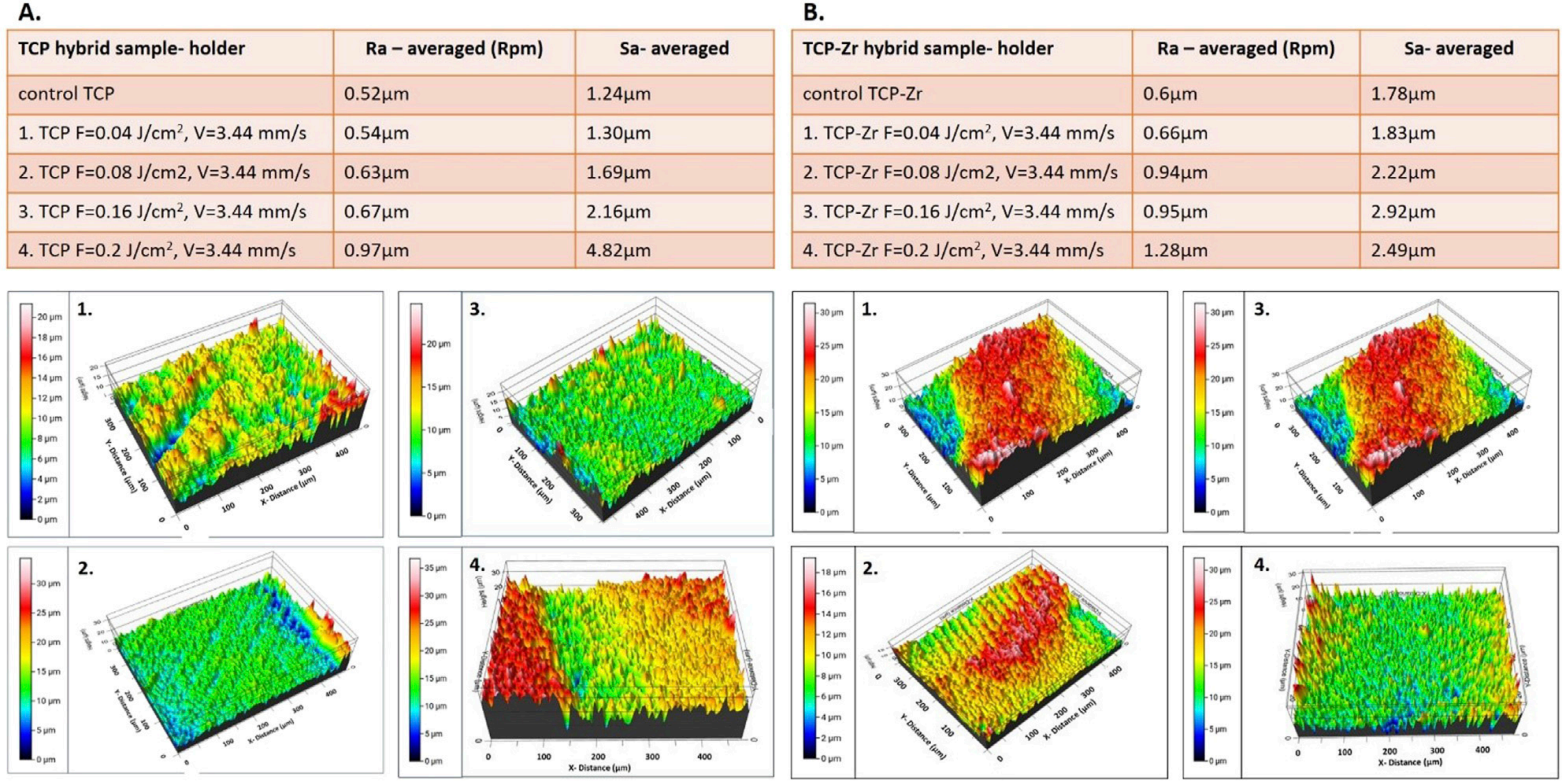
Representative 3D real color reconstruction microroughness images of the laser structured holders of **(A)**. TCP and **(B)**. TCP-Zr 3D ceramic constructs, processed at F = 0.04–0.2 J/cm^2^ and V = 3.44 mm/s; Ra and Sa roughness parameters are also presented.

Apart from the stripes created by the laser beam scanning over the surface of the material, additional microroughness in the form of protrusions, granular-like structures, and pores are also observed inside the created grooves (Figures 4, 5). This hierarchical morphology is confirmed by the roughness analysis performed - Figure 6, where linear (Ra) and surface roughness (Sa) are presented in the form of some representative 3D real color reconstruction images.

The dense structure of the support columns of the hybrid constructs, and roughness evaluation introduced by the laser structuring were performed, as the freeze-foamed bulk of both types of samples is characterized by very high (macro) porosity even before laser processing. As can be seen from the presented results, the main trend is related to the increasing surface (Sa) and linear roughness (Ra) of the support columns for both types of examined samples (pure TCP and TCP-Zr), simultaneously by increasing the applied laser fluence at constant scanning velocity. This fact could be explained by the higher laser energy deposition, which leads to more profound morphological changes and material disposal. The introduced roughness on the surface of the samples’ holders could enhance protein and cellular attachment to this inert part of the constructs which could make the scaffolds more “adhesion friendly” by promoting cellular focal contact formation (Bacáková et al., 2004; Szmukler-Moncler et al., 2004). Moreover, in almost all cases examined, the roughness parameters of the laser-treated support columns are kept under the value of 2.5 μm, which falls into the optimal range for stable bond formation of the temporal ceramic scaffold surface to recipient bone tissue (Riveiro et al., 2012; Ponsonnet et al., 2003).

**FIGURE 7:**
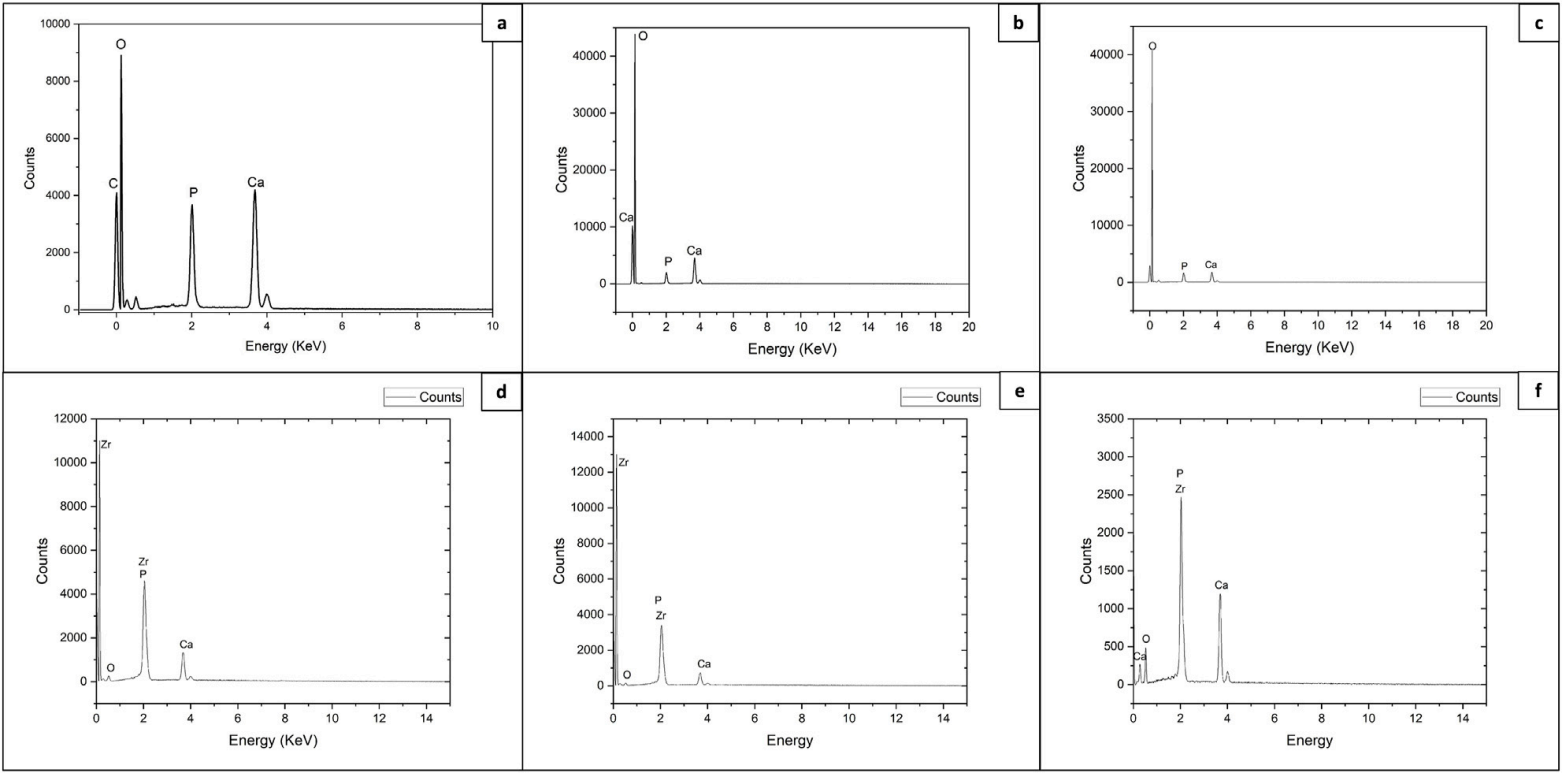
EDS analysis of control and fs laser treated 3D HAP derived TCP and TCP-ZrO_2_: **(A)** control area of TCP holder; **(B, C)** laser structured holder and bulk areas of TCP scaffolds, respectively (V = 3.8 mm/s; F = 0.08 J/cm^2^); **(D, E)** laser structured holder and bulk areas of TCP-ZrO_2_ scaffolds, respectively (F = 0.08 J/cm^2^, V = 3.8 mm/s); **(F)** laser structured holder area of TCP-ZrO_2_ scaffolds (F = 0.2 J/cm^2^, V = 3.8 mm/s).

The energy dispersive spectroscopy (EDS) performed in parallel with SEM on non-treated samples for both types of materials, and on the samples which were modified did not show differences in elemental composition. All expected elements were detected, the deviation was observed only in the intensities of the detected peaks Figure 7.

The higher laser energy density corresponds to the more pronounced appearance of the peaks of Zr and P, which could be attributed to stronger material expel and ablation. The rearrangement of the ablated material can result in its local build-up, hence, yielding a more pronounced signal during EDS.

### 3.2 XRD analysis

**FIGURE 8:**
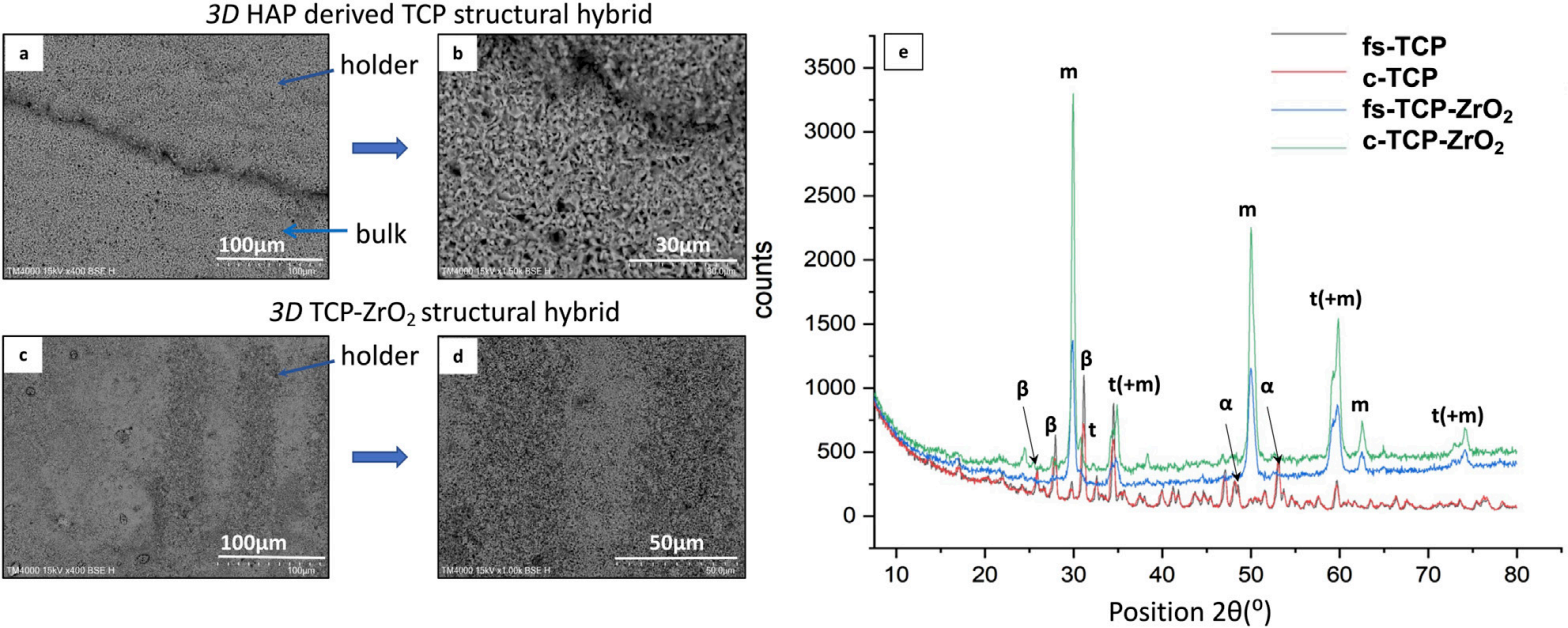
SEM images **(A–D)** and X-ray diffractogram **(E)** of laser textured 3D HAP derived TCP structural hybrid samples (fs-TCP) and 3D TCP-ZrO_2_ (fs–TCP - ZrO_2_) structural hybrid ceramics, obtained under irradiation at F = 0.04 J/cm^2^; V = 3.8 mm/s.

An X-ray diffractometry is performed to identify the crystalline phase composition. In Figure 8 are presented XRD diffractograms of the laser-processed zones concerning as-synthesized samples.

**FIGURE 9:**
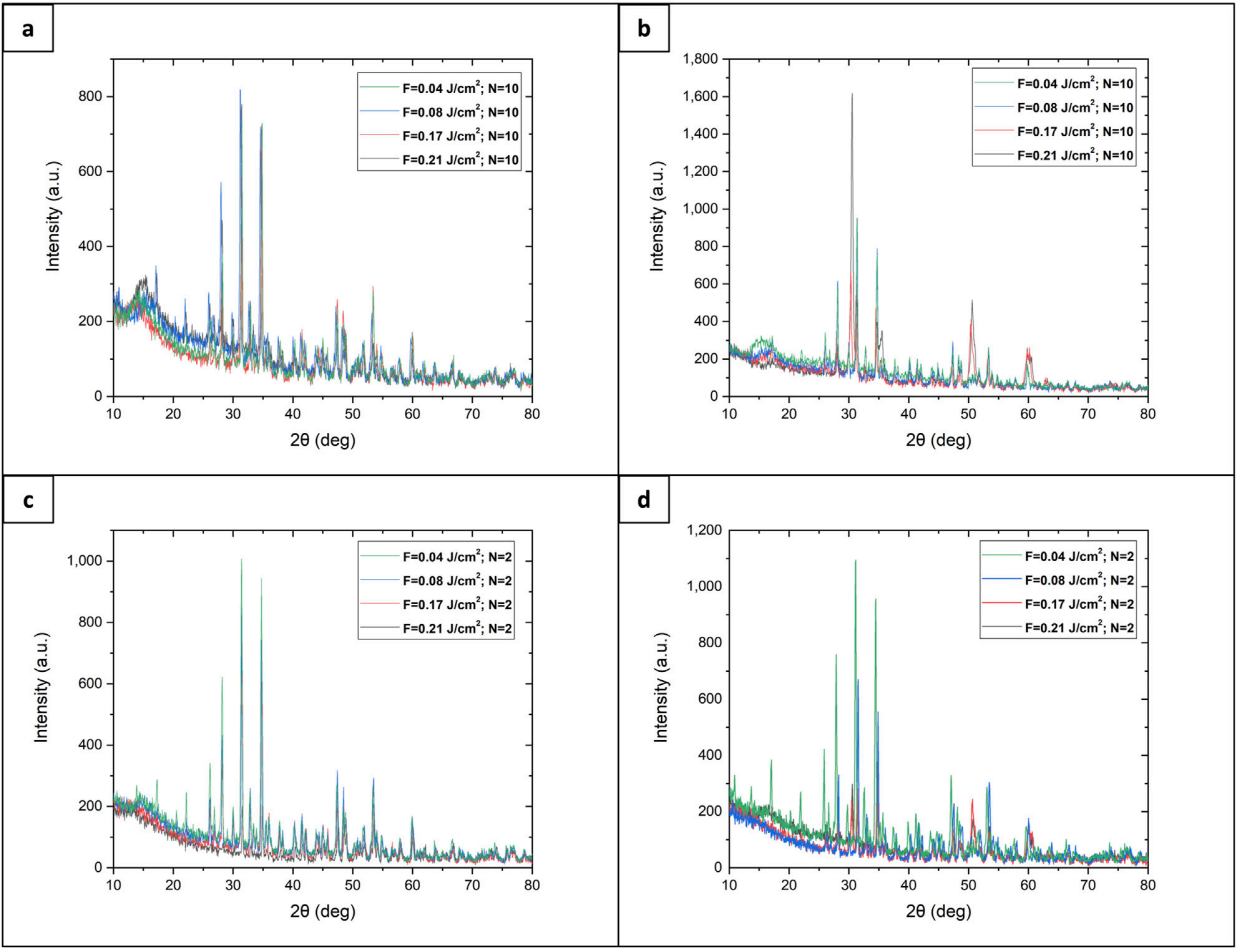
XRD spectra of laser-modified 3D HAP derived TCP structural hybrid samples (fs-TCP) and 3D TCP-ZrO_2_ (fs–TCP - ZrO_2_) structural hybrid ceramics: **(A)** TCP support structure; **(B)** 3D TCP-ZrO_2_ support structure, **(C)** TCP foam structure, **(D)** 3D TCP-ZrO_2_ foam structure.

The main diffraction peaks detected from 3D TCP-ZrO_2_ samples at 2θ = 29.85°, 34.4°, 50°, 59.25°, 62.5°, and 73° are attributed to the monoclinic phase of ZrO_2_ (m-ZrO_2_) (Mangla and Roy, 2019) - (ICDD File No. 37-1484). Whereas the peaks at 30.8°, 35°, 59.8°, and 74.1° belong to the tetragonal phase of ZrO_2_ (t-ZrO_2_). The change in crystal phase is defined by peak merging (Figure 9) towards decreasing the peak intensity of the monoclinic phase to increase the peaks’ intensity with the tetragonal phase, observed mainly on the spectrum obtained from the laser-processed sample. The reason behind such changes being noted only in the modified samples was that the femtosecond laser radiation delivers an extremely high amount of energy during the laser-matter contact which could induce a shift in the crystalline phase of the elements.

The diffractogram from 3D HAP-derived TCP structural hybrid samples exhibits the most pronounced peaks at the same position as the one for 3D TCP-ZrO_2_, with small deviations expressed in peaks with 2θ = 25.9°, 28°, 32.5°, and 47°, which are attributed to the β - phase, whereas transition to α-phase is monitored for the peaks - 48.1°, 48.5°, 53°, and 53.7° only in the case of laser processed samples.

### 3.3 Raman analysis

**FIGURE 10:**
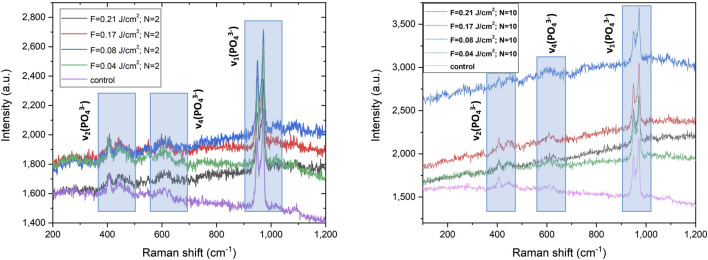
Raman spectra of the laser irradiated support structure with two different velocities (left panel) V = 16 mm/s; (right panel) V = 1.72 mm/s of the pure TCP hybrid sample.

**FIGURE 11:**
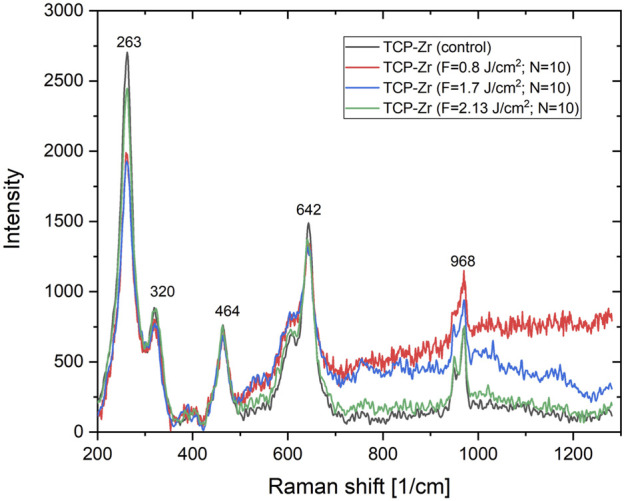
Raman spectra of the laser irradiated support structure at velocity (a) v = 1.72 mm/s of the TCP-Zr hybrid sample.

A Raman study on 3D HAP-derived TCP and 3D TCP-ZrO_2_ samples after femtosecond laser processing demonstrates negligible phase shift in the spectra - a difference only in the intensity of the detected characteristic peaks corresponding to treatments with varying laser fluence is monitored. Figures 10, 11 illustrate the acquired spectra before and after laser treatment. For the 3D HAP derived-TCP the main appearance of the peaks related to ν_1_, ν_2,_ and ν_4_ vibration mode of the phosphate group are attributed to 3 normal modes of vibration of the PO_4_, decomposed as 1A1 (ν_1_) +1E (ν_2_) + T2 (ν_4_) (Quillard et al., 2011). We monitored a slight decrease in the peaks’ intensities for spectra acquired from samples processed with lower scanning speed (V = 1.72 mm/s) Figure 10 (b). In contrast, spectra obtained for laser treatment with increased scanning speed (V = 16 mm/s) for the whole range of laser fluencies demonstrate the formation of more pronounced and shaped peaks appearing in Figure 10 (a).

In Figure 10 (a) and (b) Raman spectra of the 3D HAP-derived TCP are visible in three main peak groups expected to appear from the analyzed samples. The full set of acquired spectra is prevailed by (ν_1_) vibration mode detected between the range of 920–1,000 cm^−1^. Other groups are located in the range of (ν_2_) 390–490 cm^−1^ and (ν_4_) 550–655 cm^−1^. It is noted that the shape of Raman spectra does not change, for variation of laser fluence and scanning velocities. The split peak attributed to the vibration mode ν_1_, as well as those, attributed to ν_2_ (corresponding to E symmetry of the O-P-O bond) and ν_4_ vibration mode (corresponding to O-P-O bending) of the PO_4_^3-^ the group does not change positions, but increase their intensity after laser treatment, which could be explained by the fact that in the ionic crystal, the site and the group factor symmetries induce splitting of the internal frequencies (Quillard et al., 2011; Quillard et al., 2012).

In the case of 3D TCP-ZrO_2_ samples (Figure 11), the characteristic peaks for the tetragonal phase of ZrO_2_ are clearly outlined–maximums at 263, 320, and 642 cm^−1^ (Yashima et al., 1994). The Raman spectra of 3D TCP-ZrO_2_ show the typical TCP vibration bands, already outlined at presenting pure TCP Raman spectra, as well as vibration bands at 464, and 520 cm^−1^, which are attributed to a monoclinic phase of ZrO_2_ (Kumari et al., 2009; Kumari et al., 2008).

### 3.4 Wettability analysis versus laser-enhanced surface roughness

**FIGURE 12:**
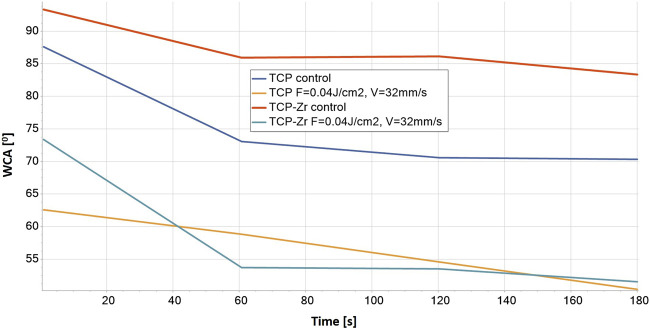
WCA evaluation of control and laser-structured (F = 0.04 J/cm^2^, V = 32 mm/s) 3D ceramic samples for a period of 180 s of dH_2_O application.

Apart from the control surfaces of the TCP and the TCP-Zr hybrid samples as well as the laser ablated TCP-Zr hybrid at lowest fluence F = 0.04 J/cm^2^ and highest scanning velocity V = 32 mm/s used in the current study (presented in Figure 12 below), all other ceramic constructs exhibited superhydrophilic properties of the laser-treated surface and the WCA was not measured, as it was not possible to be detected by the equipment due to complete spread of dH_2_O droplet upon contact with the surface. Even in the case of the results presented in Figure 12, the average contact angle values detected decrease during the 3-min measurement concerning the corresponding control surface. All measurements were conducted over the dense areas of the holders and the highly porous bulk parts were avoided due to the possibility of compromising the observation of contact angle evolution.

For the control samples, WCA values obtained decreased from around 93^ɵ^ to around 83^ɵ^ for TCP-Zr and from 87^ɵ^ to 70^ɵ^ for pure TCP constructs, indicating that the non-ablated hydrophilic surfaces become even more hydrophilic after laser ablation–the WCA decreased up to 50^ɵ^ for both laser-structured ceramic samples. Due to superhydrophilicity, statistical comparison could not be performed on all groups of samples. Only the above mentioned samples shown in Figure 12 were a subject of analysis, which showed that the differences between control and laser treated surfaces for the corresponding material (pure TCP or TCP-Zr) were significant (*p* < 0.05). This increased hydrophilicity can be explained with the Wenzel model that applies to a fully wetted surface under the water drop, which describes the homogeneous wetting of rough surfaces at a stable equilibrium state—the minimum free energy state for the system that the water drop requires. (Wenzel, 1936). This corresponds to morphology having a micro-channel structure with highly detectable roughness deviation, which is confirmed by the SEM and profilometer measurements performed (Figures 4–6) (Yilbas, 2015). The monitored transition from hydrophilic to superhydrophilic nature of most of the ceramic scaffolds (not possible to measure) could be expanded and precisely controlled by further varying the laser parameters applied (not only F and V but even fs pulse duration, laser beam polarization, and angle of incidence on the sample treated) (Wang et al., 2016). Enhancing the 3D matrix wettability could permit control over cell migration and orientation, which are crucial for natural bone regeneration and vascularization (Szmukler-Moncler et al., 2004; Rosales-Leal et al., 2010).

### 3.5 Cellular response on laser-processed surfaces

**FIGURE 13:**
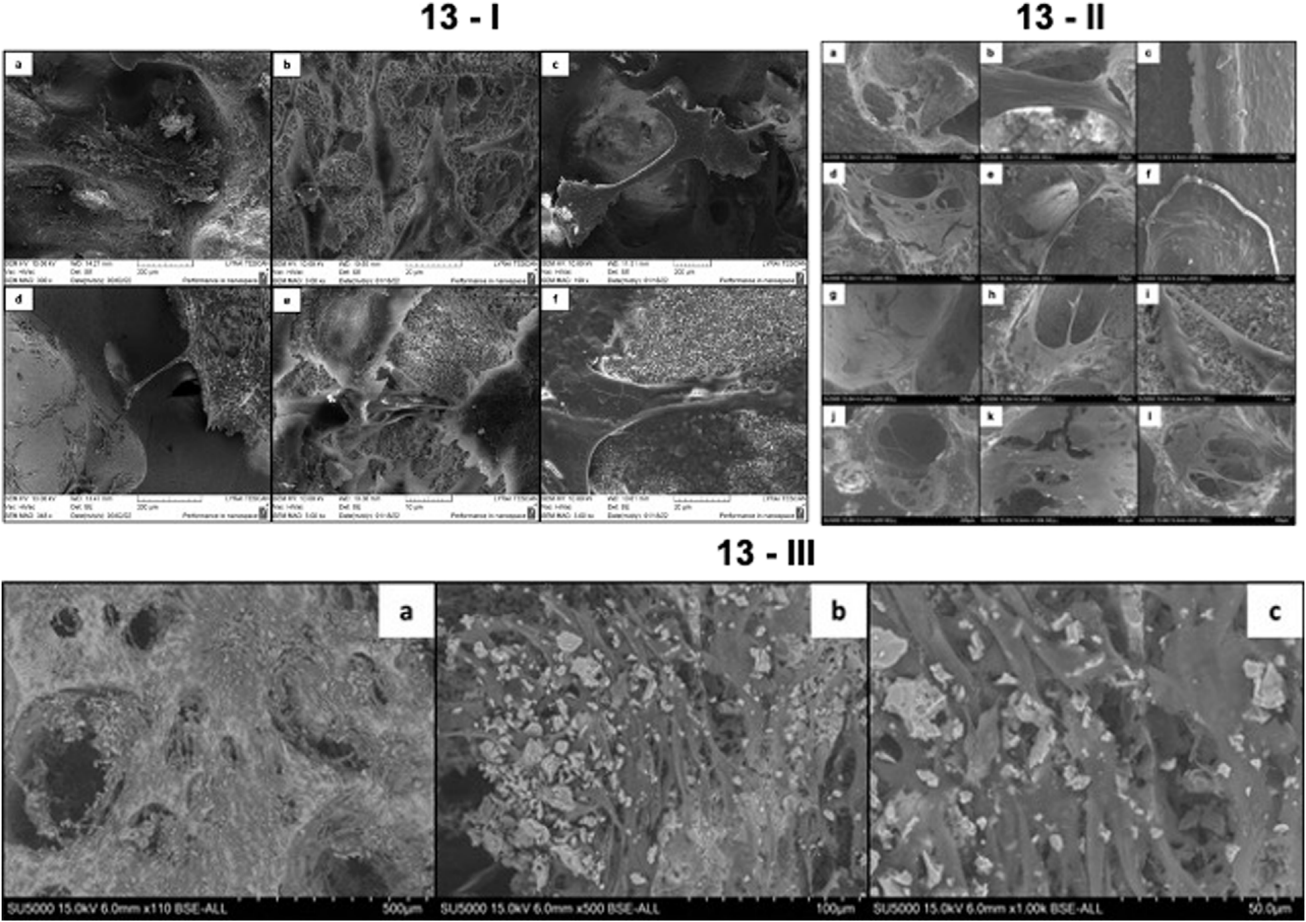
**(I)** Growth of MG-63 cells on TCP and TCP-ZrO_2_ scaffolds for 5 and 10 days of culture in basal media. (a) Control HAP-derived TCP scaffold on day 5 of culture; (b,c) laser-treated HAP-derived TCP surfaces (F = 0.08 J/cm^2^; N = 5) on days 5 and 10, respectively; (d) control TCP-ZrO_2_ on day 5 of culture; (e,f) laser-treated TCP-ZrO_2_ surfaces (F = 0.08 J/cm^2^; N = 5) on days 5 and 10, respectively. **(II)** MG-63 cell morphology on TCP and TCP-ZrO_2_ scaffolds after 7 days of incubation with osteogenic factors. (a-b) Cells spreading and forming bridges within the macropores of the fs laser processed scaffold (F = 0.08 J/cm^2^; N = 5); (c) Cells forming a continuous dense layer over the processed holder (F = 0.08 J/cm^2^; N = 5); (d-e) MG63 cells spreading inside the untreated pores of the scaffold; (f) Formation of cell layer over control area on the holder of the scaffold; (g-i) Osteoblastic cells spreading and migrating over fs laser processed surface of TCP-ZrO_2_ (F = 0.08 J/cm^2^; N = 5); (j–l) MG63 cells creating bridges over macropores inside untreated TCP-Zr scaffold. **(III)** Mineral deposition by MG63 cells after 5 days of incubation on pure TCP scaffolds. **(A)** x110; **(B)** x500; **(C)** x1,000.

**FIGURE 14:**
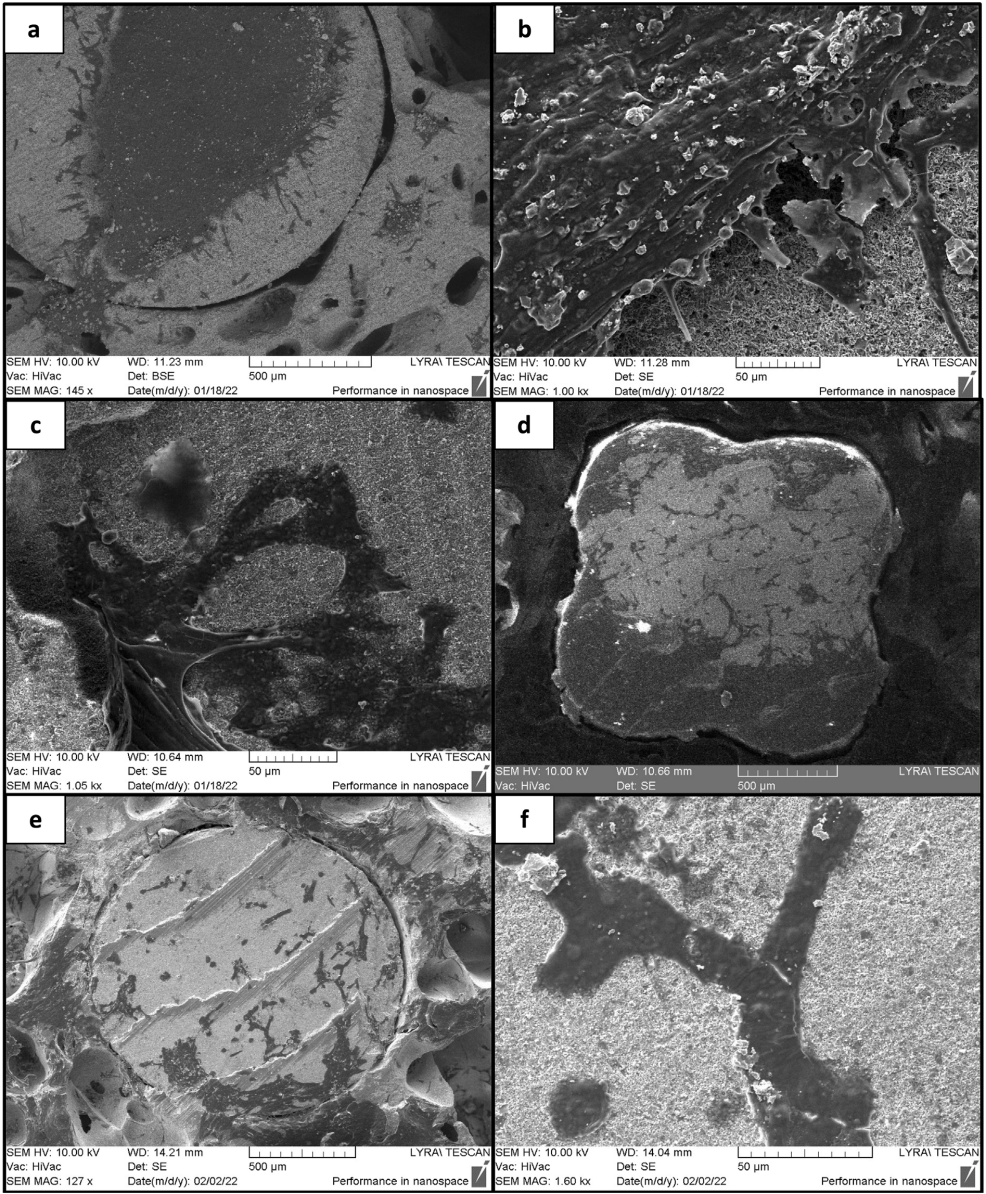
Proliferation of MG-63 cells over laser structured and control ceramic holders after 10 days of incubation. **(A, B)** Cells growing on laser-structured HAP derived TCP holder (F = 0.08 J/cm^2^; N = 5); **(C, D)** cells growing on laser-structured TCP-ZrO_2_ holder (F = 0.08 J/cm^2^; N = 5); **(E, F)** cells incubated over control TCP-ZrO_2_ holder.

Preliminary *in vitro* studies with MG63 cells were carried out. After 5, 7, or 10 days of incubation in the respective culture media on both fs laser structured and control surfaces on TCP and TCP-Zr scaffolds, the morphology of MG63 cells was analyzed via SEM. The results of both *in vitro* studies were similar as they demonstrated that the cells had spread over both treated and untreated surfaces of both types of scaffolds, exhibiting natural morphology regardless of the type of culture media (Figures 13–I, 14–II). By observing only this aspect, it could be concluded that there was no cytotoxic effect of the materials on the cells. Such observations have been confirmed before by Ahlhelm et al. (Ahlhelm et al., 2021), whose study followed the viability of MG63 cells seeded on the same type of TCP scaffold, without any surface treatments, for 10 days. The fabrication method of the scaffolds involving freeze foaming resulted in an increased microporosity with pores reaching a diameter of over 200 μm. Such large dimensions did not affect the infiltration and spread of the cells as thick bridges could be seen, connecting cells at opposite ends of a pore (Figures 13–IB, IID, F). This observation could mean that the pore size is optimal for sufficient cell growth and infiltration, which in turn would stimulate tissue regeneration more effectively. Moreover, pure TCP samples analyzed on day 5 of culture in basal media showed the presence of confluent cell layers secreting minerals on their outer surface (Figures 13–III). EDX analysis confirmed that the composition of the secreted material contained calcium, phosphorus and oxygen (Table 1). This observation could indicate that the architecture of the scaffolds could promote osteoinduction and formation of calcified bone tissue. Further analyses on osteogenic markers could confirm these preliminary conclusions.

**TABLE 1:** Elemental composition of material deposits on the outer surface of MG63 after 5 days of cell culture on pure TCP scaffolds.

Element	Weight %
O	33.7
Ca	37.9
P	17.6
C	10.8

As already mentioned, one of the main concerns about the design of the scaffolds was that cells could not attach to and proliferate on the dense holder surfaces, hence, femtosecond laser structuring was employed. The results of the SEM studies showed that the cells cultured in osteogenic media for 7 days on laser-treated and untreated holders did not show any differences in attachment. Confluent cell layers were noted on both types of surfaces which indicated that the presence of osteogenic factors has had a positive effect on their proliferation no matter the surface (Figures 13–IC, F). However, such results were not seen in the case of cells grown in basal media. Certain differences in cell adhesion and proliferation between treated and untreated areas were noted. Figure 14 displays the contrast between the areas of the laser-treated and untreated holder taken up by the MG-63 cells after 10 days of incubation. Panels (a) and (d) demonstrate that MG-63 cells have occupied large areas at the center and the edge of the laser-treated holder, respectively. In panel (e), however, it was noted that much fewer cells had adhered and proliferated. This could hint at the conclusion that the laser-enhanced surface roughness resulting in increased contact area has improved the contact between the cells and the material. Taking into consideration the results from the two *in vitro* studies, it could be stated that fs laser modeling of the holders’ surfaces could improve the cellular response alone without the use of additional stimulants such as osteogenic factors.

**FIGURE 15:**
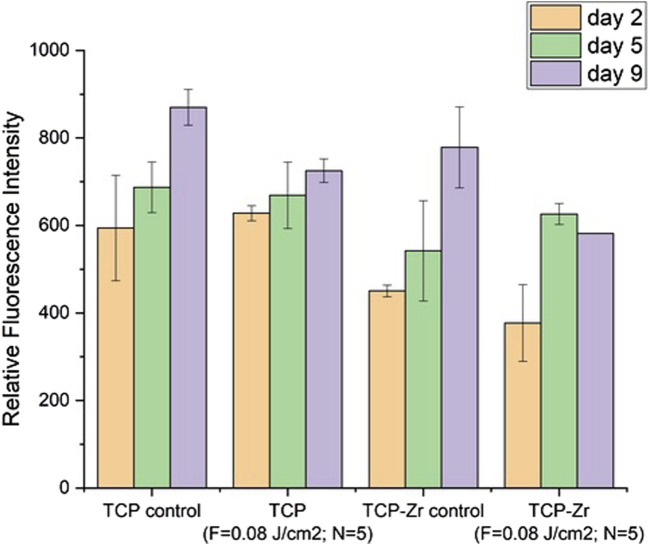
Metabolic activity of MG63 cells over 9 days after incubation on laser structured and unstructured TCP and TCP-Zr scaffolds.

Apart from the morphological analysis, the Resazurin assay was performed in order to monitor the metabolic activity of the cells for the culture period (days 2, 5 and 9). This assay could serve as an indicator for the viability of the cells. The results of the assay showed that the cells seeded on laser treated and untreated pure TCP as well as on untreated TCP-Zr samples had increasing metabolic activity as the fluorescence signal peaked on day 9 (Figure 15). The only sample which manifested a slight decrease in the signal from day 5 do day 9 was the laser treated TCP-Zr. This difference could be due to the fact that the cells had been undergoing osteogenic differentiation which could reduce their metabolic activity. Further investigation in the genetic markers for osteogenic differentiation could provide more thorough insights on these differences. Overall, it could be clearly confirmed that both functionalized and untreated scaffolds exerted positive effects on cells viability.

## 4 Discussion

In the current study three-dimensional, complex-shaped shell structures were produced by Additive Manufacturing and additionally filled with a ceramic suspension (HAP derived TCP and TCP/ZrO_2_) by Freeze-Foaming process according to previously developed and optimized procedure. In that way, highly porous 3D constructs, suitable for bone tissue regeneration were established. On one hand, these structures possess high mechanical strength due to the complex-shaped shell structures (holders), and on the other hand - very high porosity, which resembles the typical structure of bone tissue - a highly porous inner and very dense, mechanically stable outer composition. In other words, the design of the ceramic scaffolds created mimics the architecture of native bone tissue and could effectively provoke cell adhesion, orientation, and pore protrusion and induce osteogenic differentiation that could eventually lead to an improved bone healing process. Nevertheless, the weak link of the created biodegradable implants is the bioinert smooth structure of the supporting outer construction (the holders), which does not possess osteoconductive properties. Subsequent fs-laser structuring was performed to achieve optimal surface roughness and wettability of the 3D constructs, particularly the stabilizing holders. Thus, our approach provided a non-contact mode for the creation of surface characteristics that would lead to the improvement of cellular adhesion, and proliferation, by focusing on the micrometric modification of the support columns via laser-induced surface patterning. This type of surface modification has proven to be a powerful tool for the development of precise and localized structures with defined morphological characteristics (Wu et al., 2021; Ackerl et al., 2019; Carvalho et al., 2020). As mentioned before, laser-matter interaction delivers an extremely high amount of energy, resulting in the removal or modeling of material at the surface without compromising the mechanical properties of the material as a whole. Laser-induced modifications could be used to alter the physicochemical properties of a given surface either by the change in the morphology or by induction of a shift in the chemical phases of the elements (Ackerl et al., 2019). The modified ceramic surfaces were analyzed via XRD and Raman spectroscopy to monitor for potential chemical changes in the present study. The results indicated minimal phase shifts in the crystalline states of Zr as well as a slight transition from β-to α-phase of TCP. The results did not show substantial differences in the chemical states of the elements after fs laser structuring that can lead to a strong change in the chemical properties of the ceramic surfaces.

The study of the electron dynamics within the ceramic material (HAP derived TCP and TCP/ZrO_2_) is pivotal in the case of ultra-short laser-matter interaction. At the time of interaction between the femtosecond pulse and ceramic scaffold, the deposited energy is firstly absorbed by the electrons through nonlinear absorption channel. Thus, in the area of interaction it is developed a change in the absorption coefficient. The energy dissipation creates high electron density area, and increases locally the lattice temperature, generating heat-affected zones. The highly localized electron density could also induce formation of microcracks and material ablation. By tuning the laser parameters, it is possible to enhance the processing performance (by controlling the electron dynamics), altering the precision of the modification process on a micro level and processing efficiency.

Scanning velocity is one of the main factors that can influence the groove profiles, by altering the overall amount of energy accumulation at each position. Increase of scanning velocity, will lead to a less depth of the created stripes. The material ablated by a unit of applied laser energy—when the energy is deposited via a lower number of impinging laser pulses (high values of scanning velocity)—the expelled matter is less in comparison to irradiation with a higher number of laser pulses (low values of scanning velocities).

In the case of femtosecond laser-induced ablation, the distribution of laser intensity has a Gaussian profile, and the phase transition is considered to be from solid to vapor without melting. Ching-Yen Ho et al., 2011 (Ho et al., 2011), investigated theoretically the possibility for micromachining brittle aluminum oxide ceramics material with multiple pulses. According to his calculations it is possible to induce laser ablation of ceramics triggering the ablation process from melt to vapor, without incurring of melt phase. Ackerl et al., 2019(Ackerl and Wegener, 2019) demonstrated successful femtosecond laser processing of ATZ. It was found that it is possible to obtain more complex structures (micro grooves, cones and truncated pyramids, in both regimes single and multiple pulse ablation. They found that the process was highly dependent from energy density, whereas heat affected zones were found for moderate energies.

By varying the applied laser parameters, optimal structuring conditions were obtained - not only porous micromorphology was introduced, but also hydrophilic and superhydrophilic surfaces were created; According to the literature, an optimal range of surface roughness for the improved osseointegration of an orthopedic implant is considered to be between 1.2 and 2–2.5 µm (Riveiro et al., 2012; Ponsonnet et al., 2003; Yang et al., 2023) - in almost all cases examined, the roughness parameters of the laser-treated holders stay in the range of those values, which is a prerequisite for stable implantation of the construct in the recipient tissue. This, combined with the hydrophilic nature of the fs-functionalized stabilizing holders of the hybrid constructs, is the basis for improved cellular colonization, differentiation, and osseointegration. Moreover, no unwanted side chemical elements were detected and no photochemical changes in crystallinity and micro-Raman spectra were observed in the current study. As a result, no cytotoxicity was monitored. For example, the group of Li et al. demonstrated experimentally, that the femtosecond laser generation of controllable 3-D microchannels on a biodegradable scaffold can induce specific differentiation of hMSCs (human mesenchymal stem cells) *in vitro*, even without additional functionalization with biological factors (Stoilov et al., 2022), while the group of Cordero et al. showed MC3T3-E1 pre-osteoblast cell alignment on laser-formed micro patterns (Li et al., 2012). Enhancing the 3D matrix wettability could permit control over cell adhesion. It is reported in the literature, by the experimental work of Dekker et al. (Cordero et al., 2013) and Wan et al. (Dekker et al., 1991) that maximal cell attachment strength for endothelial cells and fibroblasts occurs at WCA (^0^) in the range of 20–55^0^. The monitored in the current study transition from hydrophilic to superhydrophilic wettability of the ceramic scaffolds examined could be expanded and precisely controlled by further varying the laser parameters applied. In that way, precise tuning of the optimal range of microroughness, porosity, and wettability of the biodegradable constructs could be obtained concerning the specific demands of the concrete cell line seeded. For example, the groups of E. Meurice (Wan et al., 2003) and M. Lasgorceix (Lasgorceix et al., 2018), report a tendency of cell elongation and orientation along the direction of laser-designed grooves on ceramic tablets patterned surfaces with a high increase of MG-63 proliferation after fs laser micro-patterning, compared to control ceramic substrates. The preliminary results from MG-63 osteoblast-like cell studies (Figure 13–Figure 15) did not reveal any cell alignment following the laser-induced surface microgrooves, particularly on the holder areas, but rather a mixed coverage of different areas on the surfaces. This could be attributed to the fact that the modified surface was homogenous without larger areas between separate modifications which could lead to differential attachment between smoother and rougher surfaces. Overall, SEM imaging showed a better cell affinity on the processed surfaces than the unprocessed ones and no evident cytotoxic effect on the cells. The natural cell morphology, the formation of confluent cell clusters, and the presence of cell bridging within the larger pores in the scaffolds were signs of a well-developed cell culture.

## Data Availability

The raw data supporting the conclusions of this article will be made available by the authors, without undue reservation.
